# Stretch marks: a visible expression of connective’s involvement in autism spectrum disorders

**DOI:** 10.3389/fpsyt.2023.1155854

**Published:** 2023-06-28

**Authors:** Sheila Veronese, Leonardo Zoccante, Nicola Smania, Andrea Sbarbati

**Affiliations:** ^1^Department of Neuroscience, Biomedicine, and Movement Science, University of Verona, Verona, Italy; ^2^Child and Adolescent Neuropsychiatry Unit, Maternal-Child Integrated Care Department, Integrated University Hospital Verona, Verona, Italy; ^3^Autism Spectrum Disorders Regional Centre of Verona, Verona, Italy

**Keywords:** stretch marks, autism spectrum disorder, connective tissue, tactile alteration, connectivome

## Abstract

In autism spectrum disorders (ASDs) in the pediatric population, skin manifestations are generally attributable to the concomitance of allergic forms or to accidental, self-inflicted or abusive lesions. However, clinical evidence has highlighted the presence of an increasing number of abdominal stretch marks, probably caused by the increase in the number of obesity cases in the pediatric population, in general, and therefore also among children with ASD. Stretch marks are often attributed to obesity, as they have an incidence of more than 50% in obese individuals. In the first part of this article we hypothesized that in addition to obesity there are other factors, such as a structural alteration on the skin in people with ASD, which can contribute/aggravate the phenomenon of stretch marks. Despite the high frequency with which stretch marks are found in children with ASD, this aspect has never been studied, the structure of the skin of children with ASD is not known. Furthermore, it is not known whether this structure is different from that of subjects without ASD. In the second part of the article, we hypothesized the mechanisms of the negative impact of simple abdominal stretch marks on the symptomatic picture of children with ASD. The presence of stretch marks, altered tactile perception, altered sensitivity to clothing fabrics can be a combination that influences development and determines negative consequences in the neurological picture of a child with ASD, as it is already known that the altered sensory perception in children with ASD contributes to the deterioration of social behavior. Furthermore, the presence of stretch marks may play a role in the postural and motor defects of children with ASD.

## 1. Introduction

In the context of autism spectrum disorders (ADS) in children, dermatological manifestations refer mainly to atopic dermatitis, often linked to concomitant allergic forms (1, 2), and to skin injuries (3).

As Slingsby et al. pointed out, the latter must be distinguished into accidental, self-inflicted, and abusive injuries (3). Accidental injuries are found predominantly in the lower legs such as in developing children (4), or in positions similar to those of children with cognitive and motor impairments (e.g., arms, hands, thighs, and buttocks) (5). In the latter type of children, Goldberg et al. specified that accidental injuries may be due to both natural movements, and the normal care maneuvers of caregivers (5). Self-inflicted injuries are found mainly in the hands/wrists and feet (3). Abuse injuries are of different conformation, presenting as bruises with prevalence on the torso, ear and neck for children younger than or equal to 4 years, or bruises in general for children younger than 4 months (6).

Keloids, scars, and post-inflammatory hyper- or hypopigmentation are considered secondary manifestations, often linked to self-injurious behaviors (7, 8). A high incidence of stretch marks (SMs) in the autistic pediatric population is not reported in the literature (8). However, recently, clinically, the authors have identified an increase in this incidence, a data compatible with the values reported in the literature for these manifestations in the entire pediatric population (9, 10). These data may be related to the increased rate of obesity in pediatric subjects (11), as obesity has SMs among its predominant skin manifestations in the areas of greatest fat accumulation (12, 13). The highest risk of obesity among children with ASD is already known (14–17), with causes so far identified mainly in a sedentary lifestyle and incorrect eating habits (18, 19).

An increase in obese children in the pediatric population with ASD may explain the increased evidence of SMs in these subjects. But why is this data so important? Because the authors hypothesize that these skin manifestations can be exacerbated by the alteration of the connective tissue present in the ASD (20). They also hypothesize that even from a neurological point of view there may be an aggravation of the symptomatologic picture, in particular through an overstimulation of an already altered tactile perception. Finally, they hypothesize that the presence of SMs can worsen the motor symptoms and postural instability, already described in the literature (21, 22), because they further weaken an already compromised tissue.

Therefore, in the first part of this article, a characterization of the SMs will be performed, comparing them with a typical pattern of SMs in obese subjects, but without other pathologies. In the second part of this article, the mechanisms by which they are believed to affect a child with ASD will be described.

## 2. Stretch marks

SMs has long been considered a purely aesthetic skin defect. In recent years they have been reconsidered from a pathological point of view (23).

SMs have different etiologies, because they can depend on mechanical stress of the skin (e.g., rapid weight changes, cachexia, obesity, and surgical sutures) (24–28), hormonal instability (e.g., treatments with corticosteroids, Marphan’s syndrome, and Cushing’s syndrome) (29–33), genetic diseases (e.g., Ehlers-Danson syndrome) (34), or a combination of both mechanical and hormonal changes (e.g., puberty, pregnancy) (10, 35, 36).

SMs assessment is done mainly visually. In a first phase they are defined “rubrae” and are slightly raised streaks of a pink to red erythematous color. This type of SMs is considered treatable, and there are numerous aesthetic options and numerous electromechanical tools available to prevent the degeneration of “rubrae” striae into “albae” striae (37–41). The “albae” striae are the SMs in their final stage. They have a pearly white color and are flat or depressed with respect to the adjacent skin surface. They are considered atrophic lesions of the skin (9).

SMs studies using biopsy samples showed a change in the structure of the extracellular matrix (42–45), with the reorganization of both elastic (42–45) and collagen fibers (45), and hormonal changes (46, 47). *In vivo* SMs studies, using non-invasive investigation techniques, confirmed changes in the skin’s structure (45, 48–51), and revealed alterations in its mechanical properties (35, 50, 51).

Having noticed the increase of SMs in the abdominal region in young patients with ASD, during normal clinical practice, and given the peculiarity of their shape and their distribution, the authors decided to investigate the problem further.

The literature review revealed that there are no data on SMs in ASD. There is no characterization of these skin changes in individuals with ASD. Finally, there is no comparison of SMs skin texture between subjects with and without ASD.

Randomly considered one of the days in which routine checks were performed at the Children’s Neuropsychiatry Clinic in Verona, with the consent of the parents, the photographic material of the SMs of the first two patients with ASD (of different sex) who arrived, and presented them, was collected.

The data were compared with those already available from a volunteer subject.

Here the authors report the observed results and the first considerations and advanced hypotheses.

## 3. Cases

The volunteer subject was a 20-year-old man who had had previous evaluations of his SMs (23, 52), and had given written informed consent to the use of his data for the new comparison, respecting his privacy and guaranteeing his anonymity. The previous acquisitions, measurements, and image processing had been performed in accordance with the Declaration of Helsinki. His SMs had been photographed with a simple smart phone, and evaluated using ultrasound and elastography. These last two investigations had been conducted with a MylabTM70 device (Esaote SpA, Genoa, Italy) with a 13 MHz probe. Subsequent processing had been performed using the ImageJ.JS software (National Institute of Mental Health, Bethesda, Maryland, United States).

For the collection of the photographic material of the two children with ASD, the parents gave full written informed consent. This material was anonymized before being sent for analysis and processing. Therefore, those who performed the analyzes and elaborations only knew the sex of the children and that they were two teenagers. The data was processed in full compliance with the principles of the Declaration of Helsinki. The only material collected were the photographs of the SMs obtained with a smartphone.

The control subject did not present any pathology, except for a mild obesity, believed to be the cause of the SMs. The children with ASD were obese, and had no other comorbidities. The demographics of the subjects and the characteristics of their stretch marks are summarized in Table 1.

**Table 1:** Demographics and stretch marks characteristics.

	Control subject without ASD	Children 1 with ASD	Children 2 with ASD
Demographics			
Age	20 years-old	< 18 years-old	< 18 years-old
Gender	male	female	male
Body mass index (BMI)	> 25 Kg/m^2^ & < 30 Kg/m^2^	> 25 Kg/m^2^ & < 30 Kg/m^2^	> 25 Kg/m^2^ & < 30 Kg/m^2^
Others comorbidities	No	No	No
Stretch Marks			
Shape	continuous striae, slightly raised	fragmented striae, slightly raised	fragmented striae, slightly raised
Colour	pink	dark pink	red
Length	6 cm	6 cm (longer fragment)	6 cm (longer fragment)
Width	0.5 cm	0.5 cm	0.5 cm
Number of striae in a 5 cm^2^ area (5 cm lateral to the navel)	3 vertical striae	5 vertical striae	5 vertical striae
Abrasions	no	no	yes
Sonography and elastosonography results	alteration of the connective tissue from the skin surface to the underlying muscle	not performed	not performed

**Figure 1:**
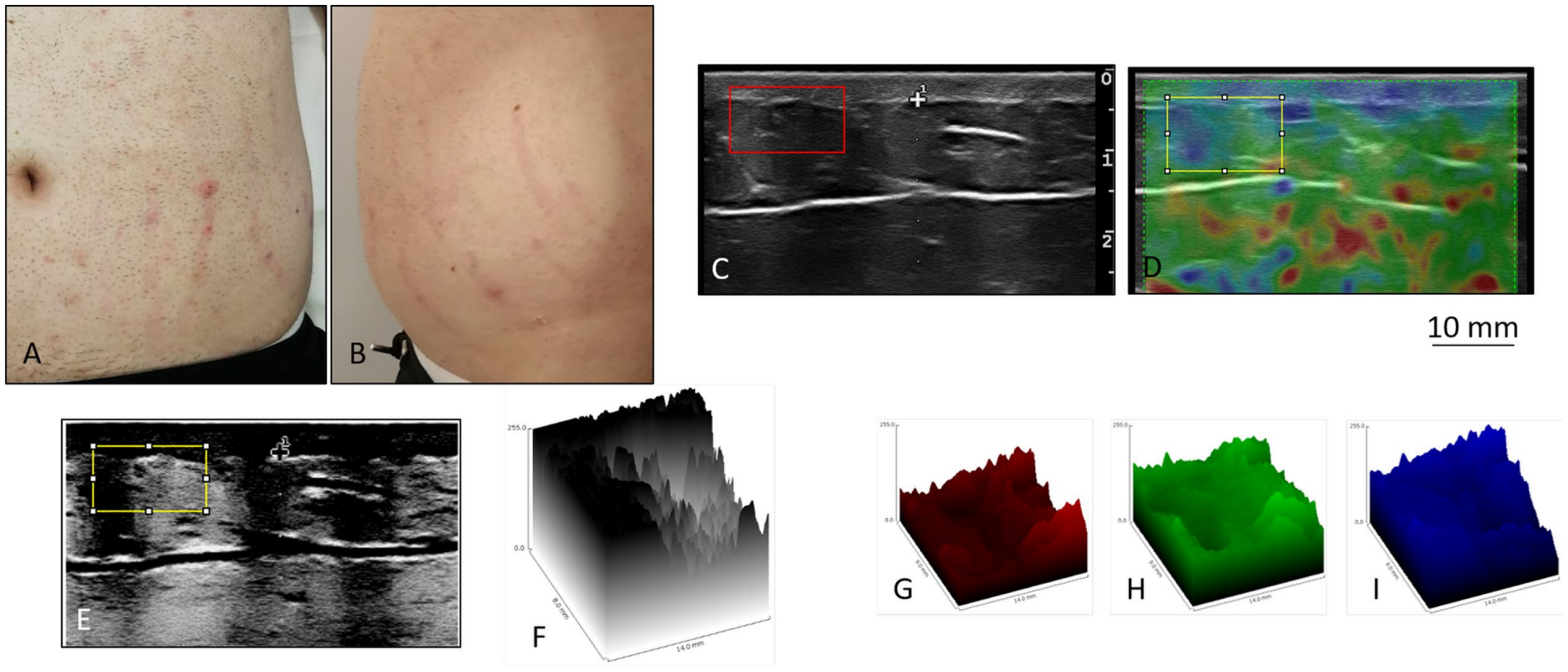
Stretch marks in a 20-year-old man. **(A,B)** The striae rubrae run vertically along the entire abdomen, from side to side. **(C)** Detail of the ultrasound. In correspondence of the SMs, columns of highly echogenic collagen fibers are highlighted. Such columns from the dermis deepen into the deep hypodermis. Original image published in Veronese et al. (23, 52). **(D)** Detail of the elastosonography. The red colour corresponds to rigid tissues, the green colour to semi-rigid tissues, and the blue colour to soft tissues. In correspondence of the SMs there are evident traces that deepen from the dermis to the hypodermis, green in colour at the level of the hypodermis. Areas of the hypodermis not underlying the SMs appear blue. In the yellow box, the left side (blue) corresponds to the intact skin, and the right side (green) corresponds to a SM. Original image published in Veronese et al. (52). **(E)** Processing of **(C)** The contrast of the ultrasound figure was increased, and the colours were reversed, in order to highlight the alterations of the connective fibers of the hypodermis in the areas underlying the SMs. **(F)** Three-dimensional projection of the yellow box in **(E)**. **(G–I)** Extrapolation of the rigid (red), semi-rigid (green), and soft (blue) components of the box selected in **(D)**. The difference between the area underneath intact skin and the area underneath a SM appears particularly evident in the semi-rigid components.

The SMs of the control subject were slightly raised, pink in color, distributed vertically along the entire abdomen (Figures 1A,B). Ultrasound and elastosonographic investigations highlighted different aspects of SMs (Figures 1C,D).The ultrasound showed the presence of areas with greater echogenicity in correspondence with the SMs, which appeared as cylinders that deepened from the dermis to the deep hypodermis (Figures 1C,E,F). Since the echogenicity described referred to connective tissue, a significant alteration in the distribution of collagen fibers was deduced, with thickening compatible with atrophy. Elastosonography revealed a different structural pattern between healthy skin areas and skin areas with SMs (Figure 1D), with the presence of a greater number of rigid (thick connective tissue), and semi-rigid (collagen fibers) components in the area with SMs (Figures 1G–I).

**Figure 2:**
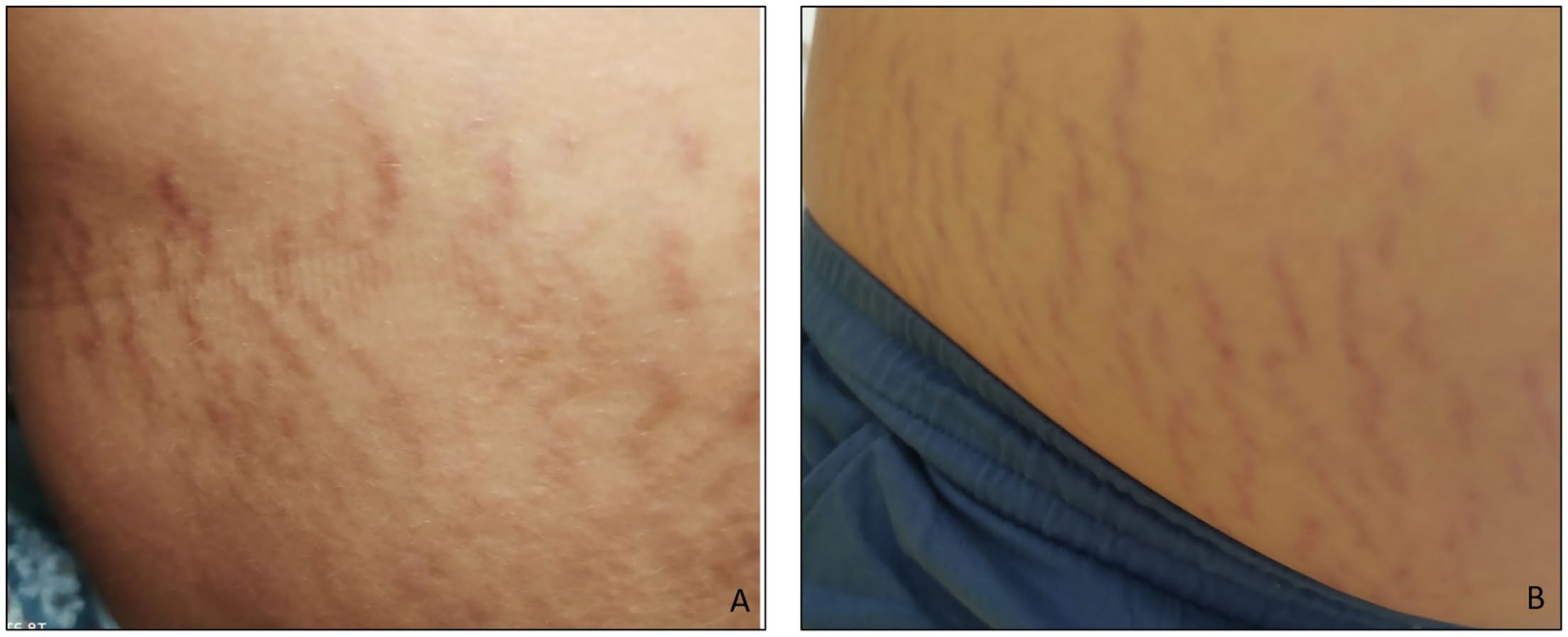
**(A)** Abdominal stretch marks in a girl and **(B)** in a boy, both with ASD. The striae rubrae run vertically along the entire abdomen, from side to side. The frequency of occurrence is higher than that observed for the subject in Figure 1, i.e., the number of SMs detected is greater.

**Figure 3:**
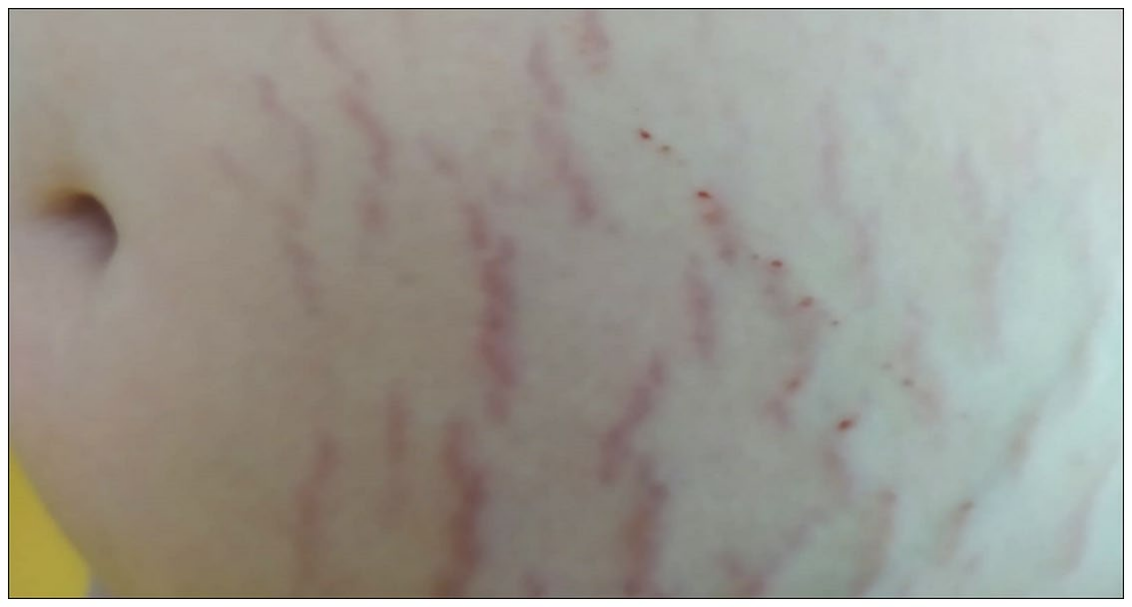
Evident scratching lesion on the right side of the boy’s abdomen.

The SMs of the two children with ASD were slightly raised, clearly redder than those of the healthy subject, indicating a more recent formation. They were distributed vertically throughout the abdomen, with a higher frequency than the SMs observed in healthy subjects (Figure 2). This means that the connective tissue alteration observed for the healthy subject was of greater severity in the two children with ASD, being more widespread. There were no apparent differences in the distribution pattern and thickness of the SMs between the two subjects with ASD, thus excluding sexual dimorphism. But it was interesting to observe scratch lesions in correspondence with the SMs of the boy with ASD (Figure 3). It is known that the SMs, in the rubrae phase, can be itchy or painful (39). However, the administration of a specific test to evaluate the presence of pain in the SMs area, and its eventual quantification, were not performed, as they were not considered routine clinical procedures, and were not yet authorized by the competent Ethics Committee.

Nevertheless, the authors wondered what could be the extent of the annoyance/pain perceived by the subject, and hypothesized the possible consequences of this annoyance/pain. Two aspects were taken into consideration: the alteration of tactile and postural perception.

## 4. Tactile perception in the ASD

In 1943, in his report on ASD, Kanner described for the first time the altered sensory perception of subjects with ASD (53). In recent years this alteration has been the subject of new studies and in particular it is proving to be an important factor in the process of deterioration of social behavior in ASD (54, 55), and all other aspects of the neurodevelopment, including detail perception, and motor planning (55).

Touch is the first sense to develop (56), and is fundamental as the first communication and attachment system between mother and newborn (56–59). Therefore, it is known that a congenital tactile alteration can considerably impair both the mother–child bond and the subsequent development of a child (55, 60, 61). Perceptual tactile alterations in children with ASD have been observed from early childhood (55, 62, 63). The changes observed by parents or examiners have appeared to be greater than effectively measurable or observed by clinicians (64, 65). Tactile sensitivity has not appeared different between children with and without ASD (66, 67), while the stimulus detection threshold was higher in children of different ages with ASD (66–68). As pointed out by Mikkelsen et al., tactile perception studies in subjects with ASD must be evaluated considering the subjectivity of the clinical evaluation, the heterogeneity of the ASD courts, and the different tactile sensitivity measurements performed (69).

However, there is no doubt that it is present an alteration of tactile perception, although obviously further studies are needed to fully understand it. Hyper and hypo-reactivity to tactile stimuli is well known (70–74). In particular with reference to the perception of vibrations, and thermal pain (71). Depending on whether the response is hyper or hypo, a different correlation with social (72, 73), and communication deterioration (72) was proven, and a different response to therapies was observed (70). The same subject can manifest reactions of different types or reactions not manifested, depending on the type of stimulus applied (75, 76). The presence or absence of response, and the type of response may also depend on the involvement of other sensory organs (75, 76). Furthermore, it should not be underestimated that being subjects with ASD, it is not possible to predict the type of response or to consider a response absent if a child does not react, as has been shown for the perception of pain (77). As suggested by Purpura et al. (55), the same stereotyped behaviors, and repetitive movements of subjects with ASD could be their compensatory systems for the inability to manage stimuli, such as tactile ones, and sensory stimuli in general. Finally, the response could also depend on the skin point stimulated, considering the different neural receptivity to stimulation of different areas of the body (78, 79).

## 5. Tactile perception of stretch marks in ASD

While SMs can be annoying in a healthy person (41), they can be even more so in a child with ASD. The excoriation presented in Figure 3 demonstrates this. Since the sites of skin lesions in children with ASD do not include the abdomen, be they accidental (4, 5), self-inflicted (3), or due to abuse (5), this excoriation can only be referred to a discomfort induced by the presence of SMs. And the discomfort could be exacerbated by the rubbing/contact of the clothing fabrics on the skin, having recently been established the different tactile pleasantness of some fabrics compared to others in subjects with ASD (80, 81).

Considering the cases presented, the itch that caused the boy’s injury is consequently attributable to the structure of the SMs. Future studies will have to clarify the causes of the alteration of the extracellular collagen matrix, extending from the dermis to the deep hypodermis. It is possible that it may be due to a number of factors combined together in a snowball effect, *in primis* caused by the general connective tissue disorder present in individuals with ASD, and well described by Zoccante et al. (20).

If in a subject without ASD, a strong weight gain determines the appearance of SMs in more than 50% of the subjects (12), the incidence of SMs in subjects with ASD may be higher, due to the presence of an already structurally different skin, i.e., more easily deformed.

Given that the maturation process of SMs from “rubrae” to the untreatable stage of “albae” is not immediate, one wonders if it is possible to intervene to “heal” the SMs in their first stage and resolve the possible discomfort.

The treatment of “rubrae” SMs is performed with various methods (37–41), more or less invasive, but not always effective. The procedures require more or less long application times and, sometimes, more sessions of treatment. It is not certain that these procedures are applicable to subjects with ASD, both children and adults, given the need for the treated subjects to remain still during the sessions. Also, not all procedures are painless. Therefore, it is not said that they can be tolerated by subjects with ASD, with altered tactile perception.

Treatments of “albae” striae are poorly documented in the literature. A technique that applies simultaneously and in synergy magnetic fields and vacuum (V-EMF therapy) seems to be very promising (82). However, even in this case it is not certain that its application can be extended to subjects with ASD.

The ideal action would be to prevent the onset of SMs. To date there are variable results regarding the topical application of creams and oils by pregnant women using these products against the onset of striae gravidarum (41, 83).

## 6. Postural and motor repercussions of stretch marks in ASD

The morpho-structural and functional deterioration of the extracellular matrix characteristic of SMs could be related to the motor and postural disturbances present in subjects with ASD (21, 22).

It is well known that childhood overweight and obesity alone primarily determine a postural alteration of the abdomen. This alteration is followed by postural imbalances of the spine, shoulder, leg and foot (84). This means general instability of the core (85).

Some studies in the literature report a correlation between striae and their severity, and prolapse of the pelvic organs (86, 87), and obstetric lesions of the anal sphincter (88). However, it is unclear whether SMs can generally be considered predictive clinical markers of urogenital dystopia (89). These data highlight the possible seriousness of the structural alteration that can be induced by abdominal SMs. Given that the morpho-anatomical alteration related to SMs affects not only the skin, but also the deep layers up to the muscles, it seems inevitable that the concomitance of SMs and the state of obesity aggravates postural alterations and core instability, followed from motor problems, which would derive precisely from these alterations. It has already been studied in children with developmental coordination disorder that improved core stability also leads to improved motor skills (90).

The already altered systemic morphology of the connective tissue present in subjects with ASD (20), alone demonstrates the correlation with postural and motor disorders. The presence of SMs and the state of obesity are aggravating factors.

## 7. Final remarks and future directions

This article is a preliminary study resulting from the clinical finding of the presence of abdominal SMs in children with ASD, SMs of different texture from the commonly known SMs in subjects without ASD. Literature is scarce on this matter, perhaps because SMs are attributed only to obesity, a known and widespread problem in subjects with ASD.

The ideas resulting from the review of the literature, considering all the possible correlated aspects, have brought out an extremely interesting picture involving both the perceptive and neurological aspects, and the postural and motor aspects.

It is evident that only hypotheses could be formulated and much clinical work needs to be done to test and substantiate them.

We have to study in detail the incidence of appearance, and the texture of SMs in subjects with ASD, in children, but also in adults. The degree of annoyance/pain that these alterations may cause should be assessed. Any behavioral impairments related to the onset and presence of SMs should be evaluated. The correlations between the presence of SMs and postural and motor alterations should be evaluated, too. Therefore, understanding if there is a worsening of alterations already present or if the mere presence of SMs causes alterations. If possible, all of these possible outcomes should be properly quantified.

Furthermore, the authors question whether what appears to be a particular distribution of SMs in children could constitute a phenotypic expression in ASDs. If this were the case, we would have a further tool for the diagnosis of ASD, especially in those borderline subjects, in which the symptoms are vague, and the disturbances are not serious. Unfortunately, given that the appearance of SMs is not premature, and seems to affect only obese children, the use of the particular distribution of SMs as a hallmark of ASD leads to a limited and, in any case, late diagnosis.

However, it should be emphasized that the fact that SMs are much more serious than in obese subjects without ASD is a clear sign of a defect already present in the underlying connective tissue, a defect present since birth. If this relevance were demonstrated, the biopsy analysis of a connective tissue sample could become a tool for early diagnosis.

In any case, the authors wonder whether and how harmful the discomforts possibly induced by SMs could be in a person with ASD. They wonder if, how much and how the structural alteration naturally present in the abdominal connective tissue and in the subcutaneous connective tissue in general can be perceived by subjects with ASD. If, how much and how the continuous contact of the fabrics of the clothes is perceived. Finally, when does this possible discomfort arise, and how neurologically harmful it can be, if present from birth, in a newborn who cannot resolve it in any way, and cannot manifest it, perhaps in any way, except by crying.

## Data availability statement

The raw data supporting the conclusions of this article will be made available by the authors, without undue reservation.

## Ethics statement

Ethical review and approval was not required for the study on human participants in accordance with the local legislation and institutional requirements. Written informed consent to participate in this study was provided by the participants’ legal guardian/next of kin.

## Author contributions

SV, LZ, and AS contributed to conception and design of the study. SV and AS organized the first draft of the manuscript and the first version of the manuscript. SV, LZ, NS, and AS performed the revision of the manuscript. All authors contributed to the article and approved the submitted version.

## Conflict of interest

The authors declare that the research was conducted in the absence of any commercial or financial relationships that could be construed as a potential conflict of interest.

The handling editor ELC is currently organizing a research topic with the authors LZ and AS.

## Publisher’s note

All claims expressed in this article are solely those of the authors and do not necessarily represent those of their affiliated organizations, or those of the publisher, the editors and the reviewers. Any product that may be evaluated in this article, or claim that may be made by its manufacturer, is not guaranteed or endorsed by the publisher.
